# The Effectiveness of Hand Massage on Pain in Critically Ill Patients After Cardiac Surgery: A Randomized Controlled Trial Protocol

**DOI:** 10.2196/resprot.6277

**Published:** 2016-11-07

**Authors:** Madalina Boitor, Géraldine Martorella, Andréa Maria Laizner, Christine Maheu, Céline Gélinas

**Affiliations:** ^1^Ingram School of NursingFaculty of MedicineMcGill UniversityMontreal, QCCanada; ^2^College of NursingFlorida State UniversityFlorida, FLUnited States

**Keywords:** massage, pain, critical care, randomized controlled trial, anxiety, muscle tension, vital signs, clinical protocol, complementary therapies, thoracic surgery

## Abstract

**Background:**

Postoperative pain is common in the intensive care unit despite the administration of analgesia. Some trials suggest that massage can be effective at reducing postoperative pain in acute care units; however, its effects on pain relief in the intensive care unit and when pain severity is highest remain unknown.

**Objective:**

The objective is to evaluate the effectiveness of hand massage on the pain intensity (primary outcome), unpleasantness and interference, muscle tension, anxiety, and vital signs of critically ill patients after cardiac surgery.

**Methods:**

A 3-arm randomized controlled trial will be conducted. A total of 79 patients who are 18 years or older, able to speak French or English and self-report symptoms, have undergone elective cardiac surgery, and do not have a high risk of postoperative complications and contraindications to hand massage will be recruited. They will be randomly allocated (1:1:1) to standard care plus either 3 20-minute hand massages (experimental), 3 20-minute hand holdings (active control), or 3 20-minute rest periods (passive control). Pain intensity, unpleasantness, anxiety, muscle tension, and vital signs will be evaluated before, immediately after, and 30 minutes later for each intervention administered within 24 hours postoperatively. Peer-reviewed competitive funding was received from the Quebec Nursing Intervention Research Network and McGill University in December 2015, and research ethics approval was obtained February 2016.

**Results:**

Recruitment started in April 2016, and data collection is expected to be complete by January 2017. To date, 24 patients were randomized and had data collection done.

**Conclusions:**

This study will be one of the first randomized controlled trials to examine the effect of hand massage on the pain levels of critically ill patients after cardiac surgery and to provide empirical evidence for the use of massage among this population.

**ClinicalTrial:**

ClinicalTrials.gov NCT02679534; https://clinicaltrials.gov/ct2/show/NCT02679534 (Archived by WebCite at http://www.webcitation.org/6l8Ly5eHS)

## Introduction

### Overview

Undergoing cardiac surgery constitutes a major event for patients that is accompanied by physical and psychological symptoms such as postoperative pain [1-5] and anxiety [6-8]. Recent studies reveal that massage could complement pharmacological treatments and have positive effects in reducing these symptoms in acute care units [9-11], yet empirical evidence is lacking to support the same effects early in the postoperative phase when patients are in the intensive care unit (ICU) and pain is at its highest.

In the ICU, postoperative pain can be compounded by routine ICU procedures such as turning, coughing, breathing, and chest tube removal, activities which are perceived to be the most painful in the immediate postoperative period [3,5]. Given the higher severity and complexity of pain in the ICU, findings from massage studies conducted on acute care wards cannot be extrapolated to the unique context of the ICU and the early recovery phase after cardiac surgery. Further evidence is needed to unravel the potential role of massage in relieving the pain of cardiac surgery in ICU patients and guide international clinical practice guidelines with regard to the use of this complementary nonpharmacological intervention in this patient population.

### Background

Cardiac surgeries, such as coronary artery bypass grafting and valve replacement, rank among the most frequently performed surgical interventions worldwide [12] and necessitate the routine admission of patients to the ICU. Cardiac surgeries are commonly indicated to reduce anginal pain, but the surgical procedure itself can lead to the development of postoperative pain. Mounting evidence shows that cardiac surgery ICU patients experience moderate-to-severe pain reaching proportions as high as 74% despite the use of analgesics [2-5], with the highest pain intensity commonly experienced in the first 24 hours postsurgery [13].

Unrelieved postoperative pain can interfere with patients’ ability to cough and mobilize effectively, which predisposes them to postoperative complications such as atelectasis, pneumonia, and deep vein thrombosis [2,5,14], thereby delaying recovery and ICU discharge. Moreover, the intensity of acute postoperative pain immediately after surgery is a significant predictor of the presence and severity of persistent postoperative pain up to 2 years postsurgery [15-17], a serious and often unrecognized complication after cardiac surgery that may interfere with daily activities and quality of life [18].

Among the pharmacological approaches to pain control, opioids constitute the mainstay of treatment in the ICU [19,20], yet pain has been shown to persist even during unrestricted use of these analgesic agents [13,21]. The use of complementary nonpharmacologic interventions such as massage has been suggested in the clinical practice guidelines of the Society of Critical Care Medicine given their opioid-sparing and analgesia-enhancing potential [20]. Massage has been defined as the manual manipulation of muscles and soft tissues of the body through the application of various systematic and rhythmic hand movements [22,23].

A recent systematic review exploring the effectiveness of massage on postoperative outcomes among patients undergoing cardiac surgery [24] indicated that out of the 7 eligible studies (N=40-252/study), 6 reported that massage therapy ranging from 20 to 30 minutes in duration improved postoperative outcomes such as pain, anxiety, and muscle tension [9,10,25-28], while only one study reported no positive results [29]. The latter study evaluated the effectiveness of a 30-minute massage therapy involving each limb for 5 minutes followed by a 10-minute back massage while patients were lying on the left side. The positioning required for the back massage might have obscured or minimized the potential benefit of massage in relieving pain, anxiety, and muscle tension given that side-lying may exert traction on the sternotomy site, causing pain [3] and increasing muscle tension and possibly anxiety as turning on one side could be perceived as a painful procedure. Of note, only one of these studies was conducted in the ICU [25], and it lacked random assignment.

One pilot randomized controlled trial (RCT) conducted with 40 ICU patients post–cardiac surgery [30] showed promising pain relief and muscle relaxant effects of up to 3 15-minute hand massages, whereas the administration of a single massage therapy did not yield any significant decrease in pain intensity or muscle tension, suggesting that repeated administration of hand massage is necessary in this patient population.

Overall, there is a paucity of high-level evidence on which to base massage therapy decisions in the management of pain in post–cardiac surgery ICU patients. Extrapolations of evidence from other patient populations and clinical settings are flawed by the differing health status and symptom severity of this specific subgroup of ICU patients. Future rigorous RCTs conducted in the context of the ICU and with cardiac surgery adults in their immediate postoperative period are essential to make recommendations for the use of massage in clinical practice including the minimal effective dose, body area massaged, and techniques employed.

**Figure 1:**
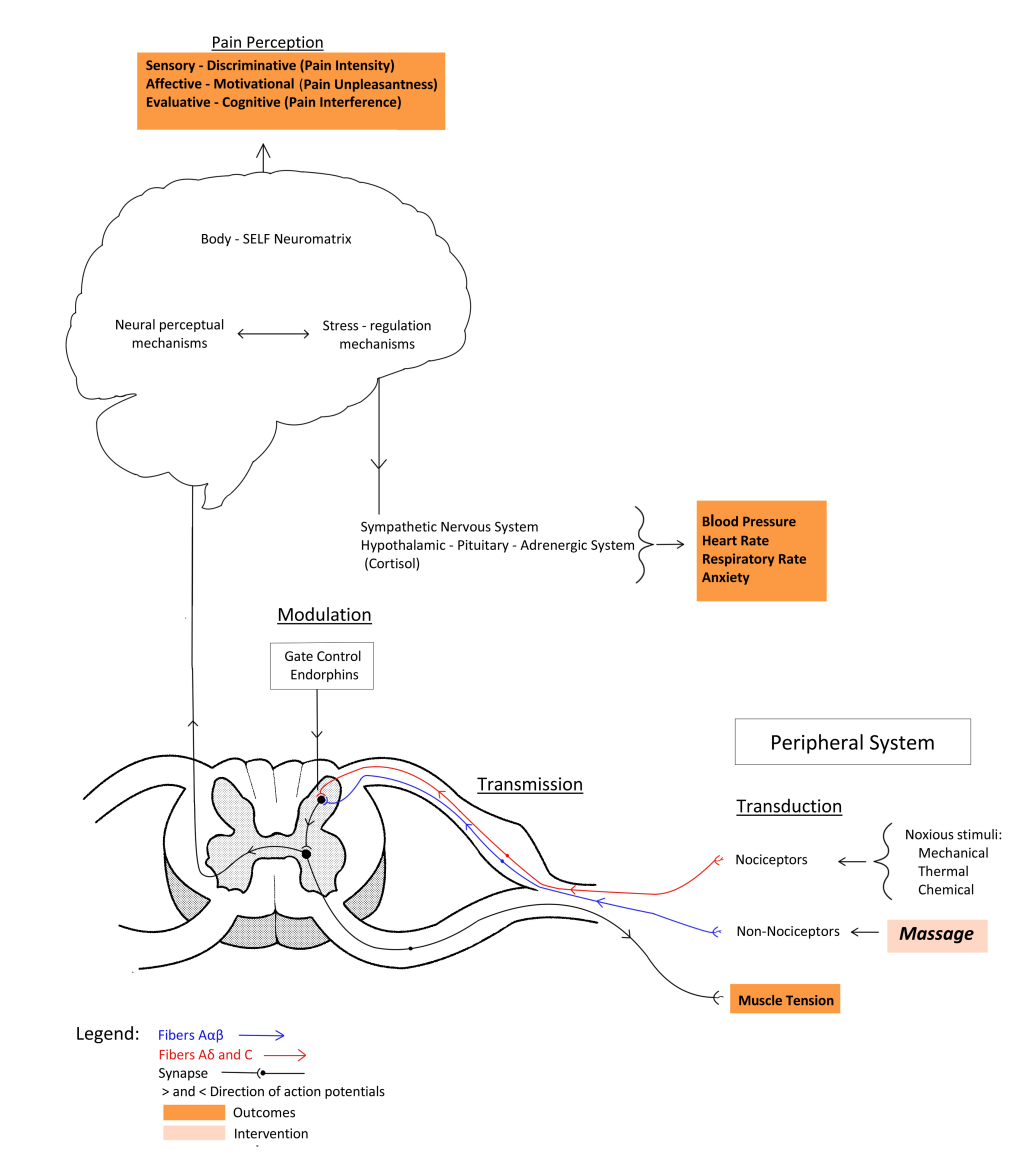
Adapted diagram of the neuromatrix theory of pain.

**Eligibility criteria.**

Inclusion criteria:

18 years of age and older

- Able to speak French or English
- Elective cardiac surgery
- Able to answer questions and self-report symptoms

Exclusion criteria:

- Previous cardiac surgery
- Diagnosis of cognitive or psychiatric disorder
- Pulmonary artery pressure >50 mm Hg
- Right ventricular failure
- Systolic left ventricular dysfunction (ejection fraction 35% or less)
- Body mass index >30 kg/m^2^
- Prolonged bleeding from the chest drainage tubes (>200 mL/h)
- Having mechanical blood pressure support (eg, intra-aortic balloon pump)
- Receiving cardiac pacing with complete control of heart rate
- Peripheral intravenous line in the hands
- Suppurating/infective/inflammatory skin condition of the hands
- Hypersensitivity to touch

### Theoretical Framework

The protocol is based on the neuromatrix theory of pain [31] where the brain’s neural network, the body-self neuromatrix, integrates multiple inputs such as sensory (eg, cutaneous) and opioid systems (ie, endogenous opioids) to influence the sensory (intensity, localization, and quality), affective (unpleasantness), and cognitive dimensions (interference with daily functioning) of pain (Figure 1). The sensory stimulation of nonnociceptive fibers in the skin and muscles involved in massage could block the transmission of nociceptive impulses in the dorsal horns and may increase the release of beta-endorphins in the bloodstream [32,33], which block the release of neurotransmitters from the nociceptive fibers (especially substance P) [34], thereby blocking the transmission of nociceptive impulses from reaching the body-self neuromatrix where pain perception occurs. Similarly, by blocking the transmission of nociceptive signals, the stimulation of the stress-regulation system responsible for the activation of the noradrenergic and sympathetic nervous system could be inhibited. The resultant lack of up-regulation of adrenaline and noradrenaline could explain the potential effects of massage in decreasing blood pressure, heart and respiratory rates, and the subjective sensations of anxiety [35-37].

### Aim

The primary aim of this research study is to compare the effect of 3 20-minute hand massage administrations by a trained nurse within 24 hours after cardiac surgery versus hand-holding (ie, simple touch) and standard care on the postoperative pain intensity of adult ICU patients.

## Methods

### Trial Design

This research study is designed as a randomized, controlled, patient-blinded, single-center superiority trial with 3 parallel groups and a 1:1:1 allocation ratio. A modified Consolidated Standards of Reporting Trials flow diagram for individual RCTs of nonpharmacologic treatments will be used to document recruitment and retention of participants (see Multimedia Appendix 1) [43].

### Participants

This RCT targets adults admitted to the ICU after undergoing cardiac surgery in a university-affiliated hospital in Canada. A single setting was selected for this study because of standardized pain management practices and surgical techniques and single patient rooms. Patients will be screened for inclusion using the eligibility criteria seen in Textbox 1. Patients at higher risk of postoperative complications and those with contradictions to having their hands massaged will be excluded.

### Sample Size and Sampling Procedure

A power analysis was conducted using the G*Power 3 program [38] to estimate the sample size required to capture the potential effects of massage to decrease the primary outcome (pain intensity) and to strengthen the statistical conclusion validity. The mean treatment difference was observed to be greater than 1.5 in several RCTs [11,26,39,40] and approximates the clinically significant difference of 2 points on the 0 to 10 Numeric Rating Scale (NRS) [41]. This trial is powered to be able to detect a difference in the pain intensity score of 1.5 between the hand massage and standard care group. The standard deviation of pain intensity scores is approximately a 2.0 value, which was selected for the sample size calculation. To detect a mean difference in pain intensity scores of 1.5 points (SD 2.0) immediately after the third massage with a 2-sided significance level of .05 and power of .80 with equal allocation to 3 arms and a repeated measures between factors context with 3 measurements would require a minimum of 72 patients.

Given the low attrition rates observed with this patient population (0% [9,10,30] and 35/287 (12.2%) [29]), a 10% drop-out rate is considered in the calculation of the total sample size. Therefore, the final size required for this RCT is 79, and it will be reached using convenience sampling.

### Randomization

Before the study begins, permuted-block randomization will be generated for 85 patients by a statistician using SAS computer software (SAS Institute) and block sizes of 3, an allocation ratio of 1:1:1, and one strata to minimize the imbalance in the number allocated to each group. Then, the randomization schedule will be transcribed in sequentially numbered and opaque sealed envelopes by a research coordinator not involved in assignment allocation to ensure allocation concealment. The allocation list will be stored in a locked filing cabinet of the principal investigator and will not made accessible to the interventionist involved in enrollment and treatment allocation.

### Recruitment of Participants

Patient recruitment will begin preoperatively when eligibility criteria such as age and language spoken will be verified (Table 1).

The remaining eligibility criteria (eg, blood loss, peripheral intravenous lines) will be evaluated post–cardiac surgery and ICU admission. After the collection of baseline data, patients meeting all the inclusion criteria will be randomly assigned to either hand massage, hand-holding, or standard care. As each participant enters the study, he or she receives the next envelope in the sequence, thereby concealing the interventionist’s and trial participants’ knowledge of the upcoming group assignment.

The interventionist will then administer the assigned intervention (hand massage, hand-holding) without informing participants of their group assignment until the end of data collection. Nurses and other ICU clinicians will also be masked to patients’ group assignment. The similarity of the hand massage and hand-holding therapy characteristics serves to mask study participants and clinicians with regard to the specific intervention received as observed in a feasibility and acceptability study where patients receiving hand-holding referred to the intervention as massage [42]. Conversely, patients in the rest group are less likely to be masked to the group assigned.

A modified Consolidated Standards of Reporting Trials flow diagram for individual RCTs of nonpharmacologic treatments will be used to document recruitment and retention of participants [43].

### Choice of Comparators

The majority of massage studies include standard care control groups to examine the absolute efficacy of massage in improving outcome variables. While this is important in attributing benefits to the massage therapy itself, studies that involve the administration of massage by a trained therapist, as recommended in Cochrane Systematic Reviews [44], should equally include a touch control group to verify if the additional manipulation included in massage is superior to touch only. Some studies suggest that touch, a free and easily administered intervention not requiring training, can have potential pain relief effects [45] and should, thus, be included as an active control group in future RCT designs to additionally examine the relative efficacy of massage. Furthermore, the touch control group can help mask patients with regard to the group assigned through its resemblance with actual massage, thereby controlling for placebo effects [44].

### Interventions

#### Training of Interventionist and Timing of Interventions

Eligible patients will be randomized in equal proportions between hand massage, hand-holding, and standard care. One interventionist will deliver the hand massage and hand-holding interventions, which will be standardized across participants. The interventionist is a registered nurse with no previous experience in massage therapy who will be trained by a professional massage therapist through an accredited workshop of 6 hours including practical exercises and verification of competency, as was done in the pilot RCT [30].

The first intervention (hand massage or hand-holding) will be delivered in the evening of the day of surgery and the remaining two interventions the day after when patients are still in the ICU. Overall, three interventions will be administered within 24 hours postoperatively over the course of two days.

#### Experimental Group

Patients assigned to the experimental group will receive a 20-minute hand massage by the interventionist in addition to the standard ICU care. Before administering the massage, a favorable environment will be created that promotes calmness such as dampening the light, reducing the alarm intensity, closing the curtains and door, and posting a “do not disturb” notice, and a comfortable positioning of the patient will be ensured [9,30,46]. After performing hand hygiene and explaining the procedure to the patient, the interventionist will hold each hand for 5-10 seconds and apply 5-10 mL of unscented hypoallergenic cream to both hands and wrists. The cream will be supplied by the interventionist and reserved for use within the research context only. The interventionist will then perform massage using moderate pressure and stroking and kneading techniques during 10 minutes on the palm and back of each hand as inspired by the procedure by Kolcaba et al [47] and developed with the support of an experienced massage therapist (Textbox 2).

**Table 1:** Study timeline.

Procedures		Preop	POD^a^ 0 evening	POD 1 early evening	POD 1 late evening	POD 2
**Recruitment**						
	First eligibility screen	x				
	Informed consent	x				
	Second eligibility screen		x			
	Randomization		x			
**Interventions**						
	Hand massage		x	x	x	
	Hand-holding		x	x	x	
	Rest period		x	x	x	
	Standard care	x	x	x	x	x
**Data collection**						
	Demographics questionnaire	x	x			
	Pain intensity		x	x	x	
	Pain unpleasantness		x	x	x	
	Pain interference					x
	Pain location and quality		x	x	x	
	Anxiety		x	x	x	
	Muscle tension		x	x	x	
	Vital signs		x	x	x	

^a^POD: postoperative day.

#### Active Control Group

The active control group will receive hand-holding by the same interventionist in addition to standard ICU care. The same hand hygiene and environmental adjustments will be made as for those receiving massage. Patients will have their hands held for 5-10 seconds and unscented hypoallergenic cream applied to both hands. Then, the interventionist will hold each of the patients’ hand in her hand for ten minutes with occasional stroking for a total of 20 minutes.

#### Passive Control Group

The passive control group will receive the standard care administered in the ICU including a 20-minute rest period including the environmental adjustments of the experimental and active control groups. The interventionist will be outside of the patient room and have no contact with the patient throughout the 20 minutes. The standard care includes the pharmacological and nonpharmacological treatments (eg, repositioning) used to promote recovery and pain relief. In the study ICU, cardiac surgery patients are automatically prescribed a pain management protocol that includes the regular administration of morphine and breakthrough doses of analgesia as needed.

### Criteria for Modifying Interventions

The allocated interventions will be discontinued upon the participant’s withdrawal of consent or if skin irritation is suspected or patient comfort is disrupted due to the intervention itself, both of which will be reported as adverse events. Consenting participants will be retained in the trial whenever possible in spite of the discontinuation of the assigned intervention to allow remaining data collection and limit missing data.

### Concomitant Care

Concomitant interventions may be received by patients while participating in this trial. Consenting patients will be permitted to receive any of the pharmacological treatments prescribed by their treating physician and any of the nonpharmacological interventions offered in the ICU (eg, back rub), and such data will be recorded. It is not prohibited that patients participate concomitantly in other research studies unless it involves any form of complementary therapy.

### Outcomes

#### Primary Outcome

Pain intensity is the primary outcome and will be captured using the 0 to 10 NRS score. The analysis metric will be the change in pain intensity from baseline (preintervention) to immediately after each intervention and 30 minutes later.

**Hand massage routine protocol.**

1. Hygiene: Wash hands with warm water.

2. Hold/connect/breathe: Make initial contact with patient, embrace hand, take a deep breath, and place feet on floor.

3. Apply cream to entire hand, dorsal and palmer aspects, including phalanges.

4. Spread (5 times) dorsal aspect of hand, using thumbs like opening a book. Repeat 5 times on palmer aspect, using the other 4 fingers to grip and glide. Spread again 5 times dorsal aspect of hand and 5 times palmer aspect.

5. Gently shake bottom hand; vibration will help the patient relax and let go of any tension.

6. Rotate wrists 3 times in each direction. Hold wrist with one hand while the other holds the patient’s hand and facilitates the passive rotation.

7. Stretch the carpal ridge 3 times along wrist crease.

8. Caterpillar walk on the dorsal aspect of the hand, between the bones (ie, zoning).

9. Pinch (dorsal/palmer) aspect of the hand in between the metacarpals.

10. Gently pull the webs of fingers.

11. Knead (rock and roll) by making a fist on the palmer aspect of patient’s hand, gently rotate using the knuckles to apply pressure 5 times.

12. Caterpillar walk on all areas of the palm, working proximally (towards the body) (if patient is less comfortable with palm upwards continue with hand in a neutral position, palm down).

13. Make small circles along the 5 zones of the palmer surface of the hand (proximal to distal).

14. Targeting key reflex areas, PRESS AND RELEASE 2 times in each area:

  a. Shoulder-neck ridge (base of fingers)

  b. Solar plexus (center of palm, just below the knuckle), PRESS and SPREAD (diaphragm line—moving outwards in both directions) 5 times

  c. Lateral aspect of palm

  d. Medial aspect of palm

  e. Pad of thumb

14. Pinch lateral aspect of hand 3 times.

15. Caterpillar walk on the medial border of thumb 3 times.

16. Caterpillar walk on the fingers:

  a. Caterpillar walk proximal making sure to cover all surface areas.

  b. Rotate knuckles gently 3 times in each direction (proximal to distal).

  c. Gently pull fingers.

17. Finishing touches: SWEEP entire hand, HOLD, and switch to other side.

18. Hygiene: Once both hands have been massaged, wash hands with cold water.

#### Secondary Outcomes

Pain unpleasantness, pain interference, muscle tension, anxiety, and vital signs will also be assessed in relation to each intervention, and means and standard deviations will be reported and used for data analysis.

### Instrumentation

#### Pain Intensity

The NRS will be used to assess pain intensity. The NRS is an 11-point unidimensional self-report scale recommended for the assessment of pain intensity where 0 is no pain and 10 the worst possible pain. Details about the NRS and other assessment tools are summarized in Table 2.

**Table 2:** Description and psychometrics of the instruments used for outcome data collection.

Outcome	Instrument	Scoring	Psychometrics	
			Reliability	Validity
Pain intensity	NRS^a^ (0-10)	0: no pain, 10: worst possible pain	High test-retest reliability observed in cancer patients when measuring pain exacerbations (kappa=.86) and background pain (kappa=.80) [48].	High concurrent validation with the Visual Analog Scale (*r*=.84 to .94, *P*<.001) [2,49]). Good discriminatory capability between background and peak intensity pain in the oncology population with only 14% of the 240 patients giving inconsistent evaluations [48].
Pain unpleasantness	NRS (0-10)	0: not at all unpleasant, 10: most unpleasant feeling possible		Good convergent validation with the Facial Affective Scale (*r*=.71, *P*<.01). Discriminant validation: correlated with the Functional Disability Inventory (*r*=.28, *P*<.05). Good sensitivity to change over a 2-week period (mean change 1.89, *t*_68_=5.30, *P*<.001) in children and adolescents after surgery [50].
Pain interference	Adapted BPI^b^: pain intensity index (4 NRS 0-10 subscales), pain interference index (7 NRS 0-10 subscales)	Pain intensity: 0: no pain, 10: pain as bad as you can imagine Pain interference: 0: no interference, 10: interferes completely	Internal consistency was also supported for this patient population with Cronbach alpha coefficients .84-.89 for the severity scale and .91-.94 for the interference scale.	Factor analysis revealed two distinct factors (ie, pain intensity and interference) accounting for 66% and 75% of total variance, respectively [51]. Scores on both scales declined significantly from baseline to follow-up, thus testifying to the responsiveness of the BPI for detecting changes [51].
Muscle tension	CPOT^c^ muscle tension subscale (0-2)	0: no resistance, 1: resistance, 2: strong resistance	Moderate to high interrater reliability of CPOT scores between trained raters with intraclass correlation of 0.30-0.86 [52] and kappa of 0.52-0.88 [53,54].	Discriminant validation: significant increases in CPOT scores during painful compared to nonpainful procedures [52-55]. Criterion validation: moderate correlation with patient self-report of pain intensity (*r*=.40-.69, *P*<.05) [52,53]. Convergent validation: moderate correlation with pain unpleasantness (*r*=.31, *P*<.01) [52]. Sensitivity of 86% and a specificity of 78% for the presence of pain during turning procedures were shown for a CPOT cut-off score >2 [56].
Anxiety	NRS (0-10)	0: no anxiety, 10: worst possible anxiety	Individual validity and reliability tests were not conducted to date with the NRS for anxiety, but it has been included in the Edmonton Symptom Assessment System, whose validity and reliability have received support over the past two decades [57].			

^a^NRS: Numeric Rating Scale

^b^BPI: Brief Pain inventory

^c^CPOT: Critical-Care Pain Observation Tool

#### Pain Unpleasantness

The NRS will be used to assess the pain unpleasantness of patients. The unpleasantness dimension of symptom experience refers to the degree to which the person is bothered by the unpleasant symptom [58]. The pain unpleasantness NRS is scored on a scale from 0 to 10 with the anchors “not at all unpleasant” for 0 and “most unpleasant feeling possible” for 10.

#### Pain Interference

An adapted version of the Brief Pain Inventory (BPI) will be used. The BPI is a short pain assessment scale developed to measure the intensity of pain during the last 24 hours (sensory dimension) and the interference of the pain in the patient’s life (cognitive dimension) [59,60]. The pain severity items are rated individually on an NRS with 0 assigned to “no pain” and 10 to “worst possible pain.” Patients are asked to rate their pain severity at the time of interview (pain now) and the worst pain, the least pain, and average pain during the last 24 hours. The 7 items of pain interference evaluate the impact of pain on general activity, mood, walking/mobilization, work, relationships, sleep, and enjoyment of life. The item work is not considered relevant in the context of cardiac surgery patients who are hospitalized in the ICU or acute care units and will not be administered. Instead, additional items about coughing, deep breathing, appetite, and concentration will be included; these have been observed to have moderate-to-severe pain interference in postoperative cardiac surgery patients [4,15,61].

#### Muscle Tension

The assessment of muscle tension will be based on an ordinal scale derived from the behavioral pain scale Critical-Care Pain Observation Tool (CPOT), which was developed and tested for the assessment of pain in critically ill patients after cardiac surgery [53]. Evaluation of muscle tension is done by performing passive flexion and extension of the upper limbs of patients at rest with a score of 0 being assigned for “no resistance to passive movements,” 1 for “resistance to passive movements,” and 2 for “strong resistance to passive movements or incapacity to complete them.”

#### Anxiety

For consistency and because intensity of anxiety is of interest in this study, the NRS will also be used to assess patients’ anxiety levels.

#### Vital Signs

Means of blood pressure (ie, systolic, diastolic, mean arterial pressure) and heart and respiratory rates will be collected from the ICU bedside monitors for 1 minute at each assessment point.

### Data Collection

Sociodemographic (eg, age, gender) and medical-surgical data (eg, type of cardiac surgery, analgesia) will be collected using standardized data collection sheets. Prior to the delivery of each intervention (hand massage or hand-holding) and before the first data collection for the standard care group, patients will complete a self-administered data collection sheet with 5 short questions using the NRS for the self-report of pain intensity, pain unpleasantness, and anxiety, a body map for identifying the site of pain, and an open-ended question for a description of the quality of pain. The interventionist will be masked to patients’ self-reports, which will be accessed only at the end of data collection to verify for missing data. The form will be completed before, immediately after the intervention (hand massage or hand-holding), and 30 minutes later, for a total of 3 data collection points per intervention. Those assigned to standard care will complete the form at similar times. This bundle of assessments will be repeated for each of the 3 interventions. Muscle tension and vital signs will also be evaluated at the same assessment points.

Finally, pain interference will be evaluated using a structured interview using the BPI on the second postoperative day to examine any carry-over effects.

### Data Analysis

The data will be entered in the SPSS software version 22.0 (IBM Corp), and a random subset of data will be used to identify missing or erroneous values. This study aims to use intention-to-treat analysis by including every participant who has been randomized, regardless of group assignment, study withdrawal, or protocol deviations.

Descriptive statistics will be calculated for sociodemographic and medical-surgical data and the baseline scores on all the outcome variables. Group differentiation in sociodemographic and medical-surgical characteristics for participant patients will be investigated using chi-square tests of independence for nominal level variables and one-way analyses of variance (ANOVA) for interval and ratio level variables. If group differences exist, the respective variable will be included as a covariate in the subsequent analyses. Frequencies and percentages of the location and descriptors of pain (ie, sensory dimension of pain) will be calculated for each group and assessment point and used to describe the pain characteristics of participants. Data on pain location and quality will be compared over time.

Repeated measures between ANOVA factors will be used to test for treatment (hand massage, hand-holding, rest), time (before, immediately after, and 30 minutes later), and interaction effect for pain intensity, pain unpleasantness, muscle tension, anxiety, and means of vital signs. This will be run for each intervention and each of these outcome variables. One ANOVA test will be performed for pain interference with the independent variable being group assignment (hand massage, hand-holding, standard care). The main comparison of interest is between the hand massage and standard care group and the difference in means pre- and immediately posttreatment.

### Ethical Considerations

Ethical approval has been granted by the Research Ethics Committee of the study setting in February 2016. This protocol has been independently peer reviewed by McGill University and the Quebec Nursing Intervention Research Network, who funded the study (F242710).

Informed and written consent will be obtained from participants. The study’s aim and procedure, risks and benefits, right to refuse participation and withdraw at any time without any repercussions on the care provided, confidentiality of data, randomization process, and lack of financial incentives for participation in the study will be explained to patients. They will be equally informed that there is no guarantee that they will benefit from this study. While there are no known risks associated with hand massage and hand-holding, any harmful effects during these interventions will be noted and reported to the Research Ethics Committee.

### Validity and Reliability

This study follows the most recent Consolidated Standards of Reporting Trials guidelines for nonpharmacological treatments and randomized controlled trials [62] and the Standard Protocol Items: Recommendations for Interventional Trials statement for clinical trial protocols [63].

The tools to be used for the assessment of the study outcomes have established validity and reliability in the population of interest in this study (Table 2). Data collection errors in vital signs will be minimized by extracting their means from the bedside ICU monitors, which offer a continuous recording of vital signs. Additionally, the interventionist will be trained on the assessment of muscle tension. To enhance rigor, standardized hand massage and hand-holding will be ensured by training the interventionist on the administration of these therapies and by using a camera to monitor the consistency and fidelity with which each of these interventions is delivered [42].

## Results

Recruitment started the end of April 2016, and to date 34 patients have already been recruited. Of these, 24 patients were randomized and had data collection done as several were medically unstable postoperatively or had their surgery cancelled/postponed. Data collection is expected to be complete by January 2017.

## Discussion

### Interpretation

Pain is one of the most common and severe symptoms cardiac surgery patients experience during their ICU stay. The adverse effects of unrelieved acute postoperative pain are numerous and can be taxing to cardiac surgery patients during their recovery but worryingly also on the long term. There is a general agreement in practice guidelines that multimodal approaches to pain management should be implemented in the ICU [20]. Massage, a complementary nonpharmacological intervention, could play an important role in enhancing pharmacological analgesia and maximizing pain relief in the cardiac surgery patients, and providing rigorous empirical evidence for its use in the ICU is strongly awaited to inform practice guidelines.

In the proposed RCT, eligible and consenting patients will be randomly assigned to either the massage (ie, experimental), hand-holding (ie, active control) or standard care group (ie, passive control). While the administration of pleasant and potentially beneficial interventions [42] can help minimize attrition rates in the experimental and active control groups, the use of standard care with rest only could cause higher withdrawals in this group and subsequently increase the risk of attrition bias. In order to counter such bias and as an incentive to make participation in the study more attractive, patients will be informed that hand massage can be offered to those assigned to the active or passive control groups at the end of data collection, if desired.

A strength of this RCT is the administration of massage in the actual ICU environment and the clinical context of the first 24 hours post–cardiac surgery when monitoring is accrued, thus enabling an evaluation of the effectiveness of this intervention on the patient’s pain and related signs and symptoms. However, the same ICU environment could interfere with the delivery of some of the planned hand massages and hand-holding in the selected mode (eg, quiet environment) and dose (ie, 3 times for 20 minutes). In order to monitor these interferences and consider them in the interpretation of study findings, a camera will be used to videorecord the interventions. Nonetheless, even if these interventions can no longer be administered (eg, use of invasive equipment on hands postrandomization), attempts to continue collecting patients’ self-reports of symptoms will be prioritized over imputation for missing data.

### Limitations

One of the anticipated limitations of this RCT is the lack of blinding of patients in the standard care group and the respective clinical personnel responsible for their care. Although environmental adjustments will be made, these patients will have already been informed of the 3 trial arms and their implications and could easily recognize their group assignment. Equally, it is expected that the ICU personnel will notice the absence of an active intervention and assume patient assignment to standard care. Given the unlikelihood of blinding related to the standard care group, patients could modify their self-report while the personnel could practice differently compared to when they see hand massage/hand-holding administrations, thereby increasing the risk of performance bias.

Only the cardiac surgery ICU patient population will be included in this RCT, which will limit the generalizability of the findings to other ICU patients. Even so, this study will reveal the effectiveness of massage in this group of ICU patients suffering from intense pain and should be a trigger for further research internationally on the use of this intervention in the ICU in addition to the locally established standard care.

### Conclusion

This funded RCT will unravel the potential benefits to reduce ICU postsurgery pain by the use of massage therapy in cardiac surgery ICU patients compared to hand-holding and standard care. The results of this study will serve to inform clinical practice guidelines with regard to the dose, timing, and technique of massage administration for the relief of acute postoperative pain in the ICU in addition to analgesia. If effective, massage could be easily implemented in ICU practice with little resources to maximize pain relief in the acute postoperative period and prevent the serious adverse consequences of unrelieved pain.

## Abbreviations

ANOVA: analysis of variance
BPI: Brief Pain Inventory
CPOT: Critical-Care Pain Observation Tool
ICU: intensive care unit
NRS: Numeric Rating Scale
RCT: randomized controlled trial

## Multimedia Appendix 1

Modified Consolidated Standards of Reporting Trials flow diagram. CONSORT E-HEALTH (V1.6).
